# Design of the INTEGRATE study: effectiveness and cost-effectiveness of a cardiometabolic risk assessment and treatment program integrated in primary care

**DOI:** 10.1186/1471-2296-15-90

**Published:** 2014-05-09

**Authors:** Ilse F Badenbroek, Daphne M Stol, Marcus MJ Nielen, Monika Hollander, Roderik A Kraaijenhagen, G Ardine de Wit, François G Schellevis, Niek J de Wit

**Affiliations:** 1 Netherlands Institute for Health Services Research (NIVEL), P.O. Box 1568, 3500 BN Utrecht, The Netherlands; 2 Julius Center, University Medical Center Utrecht, Utrecht, The Netherlands; 3 NDDO Institute for Prevention and Early Diagnostics (NIPED), Amsterdam, The Netherlands; 4 Centre for Nutrition, Prevention and Health Care, National Institute of Public Health and the Environment (RIVM), Bilthoven, The Netherlands; 5 Department of General Practice & Elderly Care Medicine/EMGO Institute for health and care research, VU University Medical Center, Amsterdam, The Netherlands

**Keywords:** Cardiometabolic disease, Prevention, (Primary) screening, Non-participation, Primary care, Family practice, Effectiveness, Economic evaluation, RCT

## Abstract

**Background:**

The increasing prevalence of cardiometabolic disease (CMD) in combination with an ageing population is a major public health problem. Early detection and management of individuals at risk for CMD is required to prevent future health problems with associated costs. General practice is the optimal health care setting to accomplish this goal. Prevention programs for identification and treatment of patients with an increased risk for CMD in primary care have been proven feasible. However, the effectiveness and cost-effectiveness have yet to be demonstrated. The ‘Personalized Prevention Approach for CardioMetabolic Risk’ (PPA CMR) is such a prevention program. The objective of the INTEGRATE study is to investigate the effectiveness and cost-effectiveness of PPA CMR, as well as to establish determinants for participation and compliance.

**Methods:**

The INTEGRATE study is designed as a stepped-wedge randomized controlled trial with a waiting list control group. In approximately 40 general practices, all enlisted patients without CMD aged 45–70 years, are invited to participate in PPA CMR. After an online risk estimation, patients with a score above risk threshold are invited to the GP for additional measurements, detailed risk profiling and tailored treatment of risk factors through medication and/or lifestyle counseling. At baseline and after twelve months of follow-up lifestyle, health and work status of all participants are established with online questionnaires. Additionally after twelve months, we will determine health care utilization, costs of PPA CMR and compliance. Primary endpoints are the number of newly detected patients with CMD and changes in individual risk factors between the intervention and waiting list control group. Medical data will be extracted from the GPs’ electronic medical records. In order to assess factors related to participation, we will send questionnaires to non-participants and assess characteristics of participating practices. For all participants, additional demographic characteristics will be available through Statistics Netherlands.

**Discussion:**

The INTEGRATE study will provide insight into the effectiveness and cost-effectiveness of PPA CMR as well as determinants for participation and compliance, which represents essential information to guide further large-scale implementation of primary prevention programs for CMD.

**Trial registration number:**

NTR4277, The Netherlands National Trial Register, 26-11-2013.

## Background

The increasing prevalence of cardiometabolic disease (CMD), including cardiovascular disease, diabetes mellitus and chronic kidney disease, in combination with an ageing population is a major public health problem. CMD mainly results from a long lasting exposure to an unhealthy lifestyle. The most important lifestyle related causes of morbidity and mortality are smoking, obesity and physical inactivity [1]. The increasing number of people with an unhealthy lifestyle is expected to lead to a rising prevalence of CMD in the coming decades [2-4]. Therefore, early detection and adequate management of individuals at risk for CMD is urgent in order to prevent future health problems and further increase in health care costs.

Screening for CMD could be more efficient when structurally embedded in primary health care [5,6]. General practitioners (GPs) can play an important role in preventing CMD [7]. General practice is the optimal setting for identifying and treating patients at risk [8]. GPs provide integrated health care, are aware of the psychosocial context and have a longstanding relationship with their patients.

Several prevention programs for CMD in primary care have been developed. These programs aim to identify patients at risk for CMD and to offer lifestyle advice and treatment when indicated [9-13]. The core elements of these programs are evidence-based and the feasibility has been positively evaluated [9-12,14-18]. Different parties have initiated implementation by offering their program to subgroups within the general population. However, the effectiveness and cost-effectiveness of prevention programs for CMD need to be established first to justify broad implementation in primary care [19]. An effective prevention program also requires structured health care, willingness to participate and compliance of patients at risk. So far, little is known about the characteristics of practices, participants and non-participants in prevention programs in primary care [20-22]. Knowledge about determinants for non-participation will support the development of tailored strategies to reach specific subgroups. In the INTEGRATE study we aim to assess the effectiveness of a CMD prevention program coupled to an individualized lifestyle intervention. This entire program will be further referred to as “Personalized Prevention Approach for CardioMetabolic Risk” (PPA CMR). Therefore, the objective of the INTEGRATE study is to investigate the effectiveness and cost-effectiveness of PPA CMR, as well as to asses determinants for successful participation in PPA CMR. In this paper we will describe the design of the study and we will discuss the choices that have been made for the intervention and with regard to outcome measures.

## Methods

### Study design

**Figure 1:**
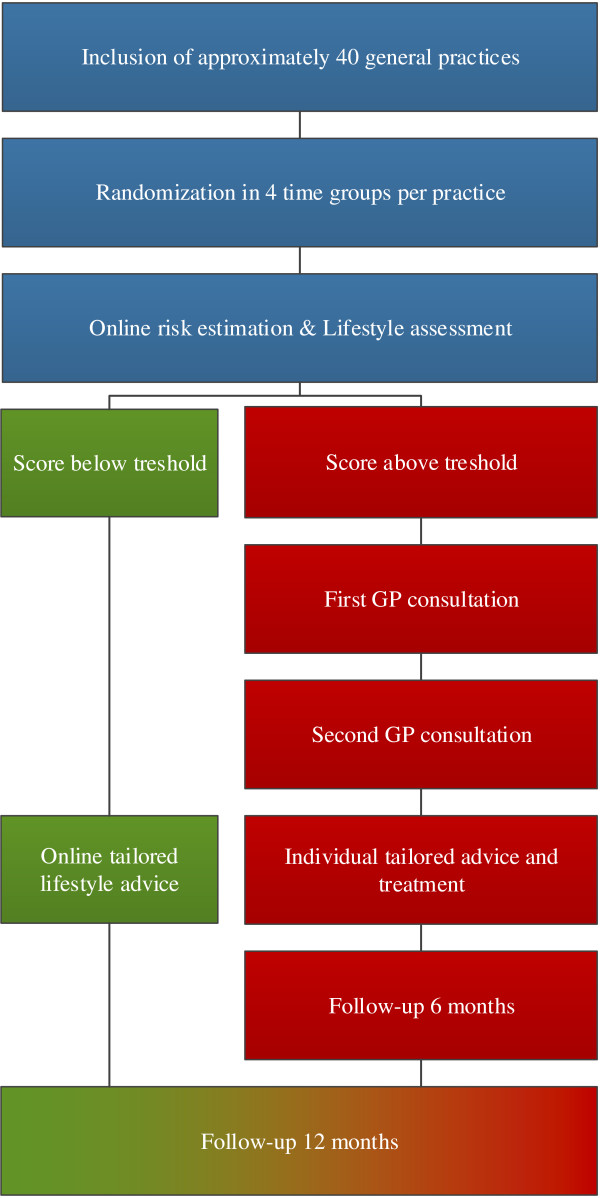
Flowchart of the study design.

**Figure 2:**
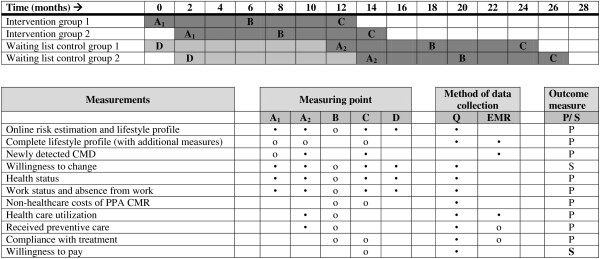
**Timeline per practice and overview of measures**. Legend: • = All patients, o = Patients with an increased risk for CMD. Q = questionnaire, P = primary outcome measure, S = secondary outcome measure.

The INTEGRATE study is a clustered stepped-wedge randomized controlled trial with a waiting list control group. A flowchart of the study and a timeline is shown in Figures 1 and 2, respectively. All participants are offered the intervention (PPA CMR) during the study period. The intervention is implemented over four time periods, in randomly ordered subgroups. The intervention group starts with PPA CMR at onset of the study, the control group starts with PPA CMR one year later. The one year waiting list period is necessary to measure natural changes in lifestyle and to estimate the number of patients with newly detected CMD without exposure to PPA CMR.

### Study population

The study will be conducted in approximately 40 general practices in the Netherlands, a representative sample of all Dutch general practices with regard to the distribution in rural/urban and solo/group practices. Inclusion and exclusion criteria for practices and patients are shown in Table 1.

**Table 1:** Inclusion and exclusion criteria for practices and participants

	**Inclusion**	**Exclusion**
**General practices**	• Use of common Electronic Medical Record (EMR) system, in which electronic data extraction is possible	• Recently performed screening for patients at risk for cardio-metabolic disease
**Patients**	• Age between 45 and 70 years	• Receiving antihypertensive or lipid-lowering treatment
		• One of the following ICPC-I-codes: K74: Angina pectoris, K75: Acute myocardial infarction, K76: Other chronic ischaemic heart disease, K77: Heart failure, K86: Uncomplicated hypertension, K87: Hypertension with secondary organ damage, K89: Transient cerebral ischemia, K90: Stroke/cerebrovascular accident, K91: Atherosclerosis, K92: Peripheral vascular diseases, T90: Diabetes mellitus, T93: Lipid metabolism disorder

Inclusion criterion for practices:

•The use of an Electronic Medical Record (EMR) system, from which electronic data extraction is possible, covering approximately 90% of all Dutch general practices.

Exclusion criterion for practices:

•Previously performed systematic CMD screening of the entire or a non-random sample of the practice population.

All eligible patients of the included practices (approximately 28.500 patients) receive an invitation letter from their GP to participate in the INTEGRATE study.

Inclusion criterion for patients:

•Age between 45 and 70 years, which is according to the guideline of the Dutch College of GPs [13].

Exclusion criteria for patients:

•Previous diagnosis of CMD according to EMR (see Table 1 for list of International Classification of Primary Care (ICPC-1)-coded diagnoses [23]).

•Receiving antihypertensive and/or lipid-lowering treatment.

### Randomization

Eligible patients are randomized within each general practice into four time groups: two intervention groups and two waiting list control groups. We will use the statistical software program Stata version 12 for the randomization. Every four months a new group starts with the intervention, starting with the two intervention groups. After twelve months the two waiting list control groups will sequentially start with PPA CMR.

### Intervention

The intervention program “Personalized Prevention Approach for Cardiometabolic risk” (PPA CMR) is the combination of a screening tool for CMD as used in the professional guideline ‘Preventive Consultation’ (PC) of the Dutch College of General Practice [13] and a tailored lifestyle intervention. PC is a Dutch prevention program for CMD and has been developed for integration in primary care (in Dutch: ‘PreventieConsult Cardiometabool risico’). In a pilot study in 2009 the PC has been tested with regard to its feasibility and was positively evaluated [8,15,17,24].

The intervention program of the INTEGRATE study consists of several steps:

1. Invitation of patients to assess their CMD risk.

2. First step of screening: the online risk estimation and lifestyle assessment.

3. Second step of screening: completing the CMD risk profile with additional measurements.

4. Treatment of patients with an increased risk for CMD with tailored lifestyle advise and/or medication.

#### Invitation of patients

All eligible patients receive an invitation from their GP to participate in PPA CMR by completing an online risk estimation and optionally an online lifestyle assessment. To enhance participation rates, the accompanying information letter will summarize the details of the study in different languages. In case of non-response, a reminder letter is sent after two weeks. Enclosed with the reminder letter is a paper version of the risk estimation.

#### The risk estimation and lifestyle assessment

The risk estimation is based on the widely accepted FINDRISK score and is specified for predicting CMD in the Dutch population [25,26]. This seven item-questionnaire can be completed by self-report and assesses cardiometabolic risk factors including age, gender, body mass index, waist circumference, current family history of cardiovascular disease and/or diabetes [13,26]. The lifestyle assessment consists of questions involving smoking, physical activity, dietary patterns and willingness to change lifestyle [9,12].

The threshold in the risk estimation that will be used is an absolute risk for developing CMD in the next seven years of ≥ 23% for men and ≥ 19% for women [26]. Patients with scores below the threshold are at low risk and receive online tailored lifestyle advice based on the reported risk factors and the information provided in the lifestyle assessment. All patients with scores above the threshold are advised to complete their final risk profile with additional measurements, by making an appointment at their general practice.

#### Completing the CMD risk profile

At the general practice, the risk profile is completed by additional measurements: serum cholesterol level, fasting glucose level and blood pressure. During a second visit the final risk profile is calculated based on the SCORE risk estimation [27].

#### Treatment of patients with an increased risk for CMD

Patients will receive treatment according to their risk profile, based on recommendations on lifestyle advice and drug treatment from guidelines issued by the Dutch College of GPs (including guidelines on cardiovascular risk management, obesity management and diabetes mellitus). Participating practices offer lifestyle interventions in their own conventional manner, with the facilities available to them. Possible facilities for lifestyle interventions include the aid of a lifestyle coach to support active lifestyle change, offering structured programs for smoking cession services, weight management or exercise programs and collaboration with other local initiatives in health programs.

### Control group

Patients allocated to the waiting list control group receive an invitation from their GP - at the same moment the first intervention group is invited- to participate in a health study by completing an online questionnaire including the questions of the risk estimation and lifestyle assessment. However, these patients neither receive a risk score, nor a specific lifestyle advice. These patients will start with a one year waiting period, to be used as control comparison. After a year they are invited to participate in PPA CMR, starting with completing the risk estimation and lifestyle assessment online. Hence, the waiting list control group is offered the identical route as the intervention group. Patients in the waiting list control group receive normal standardized care during the waiting period, including lifestyle advice or diagnostics and treatment for CMD when indicated.

#### Response-enhancing strategies

During this study we will develop and evaluate different response-enhancing strategies in subgroups of the waiting list group. The response enhancing strategies are adjusted according to the results of non-response analyses performed early in the study (see next paragraph, endpoint 5). Possible strategies include reminders by telephone, translated questionnaires for non-Dutch speaking patients, information gatherings at the general practice and verbal reminders by the GP.

Another strategy is using a toolbox to complete the final risk profile at home. It offers the option to bypass one or both of the GP consultations. The toolbox contains a blood pressure device and a laboratory test form. Patients are asked to measure their blood pressure, visit the laboratory and to complete the results online. In case of a high blood pressure and/or elevated serum cholesterol or glucose levels, patients are advised to consult their GP. Patients without elevated biomedical risk factors receive an online tailored lifestyle advice and will therefore bypass both GP consultations. Like the other response-enhancing strategies, the toolbox option will be implemented during the intervention period of the waiting list control group.

### Endpoints and measurements

The endpoints of the INTEGRATE study are shown in Table 2. An overview of all measurements is shown in Figure 2.

**Table 2:** Primary and secondary endpoints

**Primary endpoints**	**Secondary endpoints**
1. The number of newly detected patients with a CMD in one year follow-up	1. Difference in primary outcome 5 after implementation of different response-enhancing strategies
2. Change in individual risk factors (smoking, physical inactivity, obesity, unhealthy diet, blood pressure and cholesterol levels) for CMD between baseline and one year follow-up	2. Change in willingness to change lifestyle between baseline and one year follow-up
3. The expected number of newly detected patients with CMD and mortality after 5, 10,20 years and lifetime	3. Change in health status between baseline and one year follow up
4. Costs-effectiveness of PPA CMR	
5. Non-participation and compliance in different stages of PPA CMR	

For our secondary endpoints we will use the information provided for our primary endpoints.

1. *Newly detected patients with CMD at baseline and one year follow-up*

The number of newly detected patients with pre-existing CMD will be established after the second consultation and after one year follow-up, based on ICPC-1-coded diagnoses (see Table 1) in the EMRs.

2. *Change in individual risk factors for CMD between baseline and one year follow-up*

For patients with an increased risk for CMD, risk and lifestyle profiles will be established at the start of PPA CMR and after twelve months of follow-up. Risk profiles consist of the completed risk profile including the additional measurements done by the GP or with the self-management toolbox. The questions of the online risk estimation and lifestyle assessment are repeated after six months as well (see Figure 2).

For patients with a low risk for CMD we will establish risk and lifestyle profiles at the start of PPA CMR and after twelve months of follow-up. These risk profiles do not contain the additional measurements.

3. *Expected newly detected patients with CMD and mortality after 5, 10, 20 years and lifetime*

We will use the RIVM-Chronic Disease Model (RIVM-CDM) [28,29] to extrapolate the number of possible prevented CMD due to PPA CMR with a time horizon of 5, 10 and 20 years. The calculations are based on changes in risk profile during one year of treatment.

4. *Costs-effectiveness of PPA CMR*

For patients with an increased risk for CMD, we will establish health status, work status and absence from work at the start of PPA CMR and after six and twelve months of follow-up. Health status is measured by the validated Dutch version of the SF-36 [30] and the EQ-5D [31,32]. Work status and absence from work is measured by using parts of the Productivity Cost Questionnaire (iPCQ) [33].

Healthcare and non-healthcare costs are measured after six and twelve months of follow-up. Healthcare costs include the costs of implementing PPA CMR and any lifestyle intervention or treatment that emanates from the use of PPA CMR. Other healthcare costs are the costs of health care utilization during the one year follow-up. These costs are based on standard prices for health care use [34]. Non-healthcare costs include expenses made by participants during the study, e.g. own expenses for lifestyle interventions. Data on health care use, needed for the economic evaluation, will be extracted from EMR’s of GPs.

For patients with a low risk of CMD we will establish health status, work status and absence from work at the start of PPA CMR and after twelve months of follow-up.

After completion of PPA CMR, the willingness to pay for (parts of) this program is evaluated in all participants.

5. *Non-participation and compliance in different stages of PPA CMR*

Participation rates in the different phases of PPA CMR are measured by establishing the number of participants and the number of eligible patients in each stage (after the first invitation, after completion of the online risk estimation, after completing the risk profile and during the treatment phase). Data about the numbers of participants in each phase can be derived from the website for online respondents. The number of practice visits and compliance with treatment is established at six and twelve month with data from EMRs and self-reported compliance. We will collect information on determinants of response and non-response through the use of three different sources. First, we will send questionnaires to a random sample of patients who did not respond to the invitation of their GP for participating in PPA CMR (non-response group 1). This non-response questionnaire contains items on health risk behavior, assumptions about CMD and screening, reasons for not participating and attitudes towards response-enhancing strategies (see Table 3). In addition, we will send a comparable online non-response questionnaire to patients who scored above the threshold on the online risk estimation, but did not consult their GP (non-response group 2). Second, we will extract anonymized data from EMRs, including information on health care utilization of both participants and non-participants. Finally, all data will be linked with data from Statistics Netherlands to obtain information about socio-economic status (SES) and ethnic background.

**Table 3:** Overview of measurements among non-responders

**Non-response questionnaire**	**T=0**	**T=12**
Risk estimation (paper)	*	
Online risk estimation and lifestyle profile	o	o
Attitude towards screening and treatment of CMD	•	
Reasons for non-participation	•	
Attitude towards response-enhancing strategies	•	
Newly detected CMD (EMR)		•
Health care utilization (EMR)		•

* = Non-responders group 1 (no response to invitation PPA CMR, no online risk estimation).

o = Non-responders group 2 (score above threshold on risk estimation, but not no GP consultation).

• = All non-responders (group 1 + 2).

Information on determinants of non-participation and successful completion of PPA CMR is used to study the differences in characteristics of responders and non-responders. We will also study differences in characteristics of participating practices (e.g. urban/rural locations, solo/group practices, organization of lifestyle interventions) to find practice-related factors that are associated with participation and compliance rates. The analyses of determinants for participation shall be performed in the first groups starting with the intervention. Depending on the findings, response-enhancing strategies are developed and implemented in the waiting list control groups that subsequently enter the study. Data collection for subgroups receiving a response enhancing intervention is done in the same way as described above.

#### Waiting list control group

From the waiting list control group we establish risk profiles, lifestyle assessment, health status, work status and absence from work at baseline and again at the start of PPA CMR one year later. At the start of PPA CMR newly detected patients with CMD will be established, based on ICPC-1-coded diagnoses in the EMRs. Patients who develop a new CMD - documented through an ICPC-1-coded diagnoses in the EMR - will not be eligible for participation in PPA CMR, but will receive questionnaires for follow-up. When the waiting list control group starts with the intervention phase, the patients follow the identical route as the intervention group (see Figure 2).

### Analyses and statistical methods

We will analyze the data from this study according to the intention-to-treat principle. Analyses will be performed with all data available. Since the availability of data will depend on the response rate, a fully complete dataset cannot be expected. Multiple imputation techniques are used for handling missing data.

#### Sample size calculation

Calculation of the sample size is based on the reduction of smokers in the intervention group after one year follow-up, one of the primary outcome measures. The smoking prevalence in the Netherlands is 25% [35]. We expect a reduction in smoking prevalence from 25% to 20% after one year treatment and a stable number of smokers in the waiting list control group. In order to achieve this reduction, 721 patients are needed in the intervention group. This calculation is based on an alpha of 0.05 (two-sided), a power of beta = 0.80 and a ratio intervention group versus control group of 1:4). The 1:4 ratio represents a fair comparison between the intervention and the large control group. Based on the pilot implementation study of the PC, we expect approximately 21 patients per practice in the intervention group after twelve months follow -up [14,15]. A low response rate has been taken into account with this estimate. This would result in the inclusion of 721/21 = 34 general practices. However, in this study patients are clustered within general practices and an oversampling of 15% is needed to correct for this clustering in multi-level analyses. Therefore, we need approximately 40 general practices. The number of participants and practices will result in sufficient power to establish statistically significant differences between other subgroups.

#### Effectiveness of PPA CMR

We will use multivariable multilevel regression analyses to study the effects of PPA CMR on change in individual risk factors and lifestyle and on the incidence of CMD after one year follow-up. Therefore, we compare the intervention group with the waiting list control group. In addition we will evaluate the influence of different response enhancing strategies on the effectiveness of PPA CMR. We will use linear or logistic regression for continuous or dichotomous data, respectively. Multilevel analysis is needed to correct for clustering of patients within practices.

#### Cost-effectiveness of PPA CMR

We will perform an economic evaluation to relate net incremental costs and effects of PPA CMR compared to the waiting list control group. Estimated costs are based on the healthcare and non-healthcare costs. After one year of follow-up, cost-effectiveness of PPA CMR will be established. To evaluate cost-effectiveness in the long term, modeling is required. We will use the RIVM-Chronic Disease Model (RIVM-CDM) to perform this long-term economic evaluation. The RIVM-CDM is a Markov-type, dynamic population-based model [28,29] and is able to relate changes in prevalence of risk factors to changes in future incidence of CMD. The model also contains data on costs of cardiovascular events and associated losses in quality of life. This model has extensively been used for the evaluation of cost-effectiveness of prevention programs targeted at lifestyle improvement [34,36-38].

The cost-effectiveness will be calculated per level of change in individual risk factors. Incremental cost-effectiveness ratios (ICER) are derived from calculating the net costs of PPA CMR compared to the waiting list control group, divided by its effect. In addition, we will calculate the incremental cost-utility ratios (ICUR). Therefore, the incremental costs of PPA CMR compared to the waiting list control group will be divided by the effects in quality adjusted life years (QALY’s) gained. Utility values as incorporated in the RIVM-CDM will be used for future cardiovascular events. Probabilistic sensitivity analysis are performed for all calculations.

#### Determinants of participation and compliance

The number of participants during the different phases of PPA CMR will be presented with frequency tables. Differences between participants and non-participants regarding age, gender, SES, ethnic background, and cardiometabolic risk are determined using univariable analysis (*t*-test, chi-square test). We will use descriptive statistics and multivariable regression analyses to determine the profile of participants and non-participants in PPA CMR.

### Privacy and informed consent

To ensure privacy of the patients, the participating practices will send the invitation letters to the patients. Additional information in the invitation letter will inform the participants about the study purposes. At the start of the online risk estimation and lifestyle assessment, all patients are asked to complete a digital informed consent form.

We will obtain data on health care utilization of all patients through data extraction from the EMR of the GPs. Based on the Dutch law for data protection, obtaining informed consent for this part of the data collection is not necessary. All obtained data will be processed anonymous, not traceable to individual patients. The study was considered by the UMC Utrecht Institutional Review Board and exempted from full assessment under the Medical Research involving human subjects Act.

## Discussion

This manuscript describes the design of the INTEGRATE study, a study aiming to establish the effectiveness and cost-effectiveness of a Personal Prevention Approach for cardiometabolic risk (PPA CMR) in primary care. An additional aim is to provide more insight into the profile of participants and non-participants and the effectiveness of the various components of the program. Our final goal is to contribute to the reduction of cardiometabolic morbidity and mortality in an aging population.

### Choices in study design

In the design of this study we made a number of choices that need to be addressed:

1. Design

We have chosen a stepped-wedge randomized controlled trial design. Patients will either be allocated to the intervention group or the waiting list control group that starts the intervention after one year. The waiting list control group is necessary to measure ‘natural’ changes in lifestyle among eligible persons and to estimate the number of newly detected CMD without exposure to PPA CMR. At the end of the study PPA CMR is completely implemented in all participating practices and all eligible patients have received the intervention. Implementation of PPA CMR is done in time periods to distribute the workload for the GPs and their staff.

2. Randomization

Participants are not informed about the existence of a waiting list control group and none of the participants will know to which group they are assigned. Nevertheless, the nature of this intervention makes total blinding of the participants impossible. To minimize bias and maximize the validity of the results, both groups will receive the same standardized care, according to the evidence based practice guidelines issued by the Dutch College of GPs. For practical reasons, selection and randomization of all eligible patients will be done at baseline. Randomization is performed at individual level and is done to equally distribute correlating factors of patients registered within the same practice. Because randomization takes place before consenting to participate, selective response can be induced (see ‘possible methodological threats’). Randomization within practices can cause ‘contamination’, lifestyle changes of patients may affect the lifestyle of their spouse and others in their environment. When spouses are assigned to different groups this can influence the results, causing an underestimation of the effectiveness of PPA CMR.

3. Integration in routine primary care

Since PPA CMR is based on a Dutch GP guideline and can be considered ‘standard care’, we have chosen to implement PPA CMR into routine primary care. This way we can evaluate the effects of an existing screening program for patients at risk for CMD combined with tailored treatment for risk factors in the most natural way.

4. Practice characteristics

Lifestyle interventions may differ between general practices. For example, some practices have a lifestyle coach or collaborate with local providers of lifestyle interventions whereas in other practices GPs only give lifestyle advice. Changes in lifestyle are hard to accomplish, especially maintaining a healthy lifestyle asks a lot of perseverance from patients. Intensive support by a lifestyle coach or providing local lifestyle interventions may provide the necessary continuity to achieve a more sustainable reduction in cardiometabolic risk. We will carefully document practice characteristics to evaluate which factors influence compliance with and enhance effectiveness of the program.

5. Modeling

One year of follow up will not be sufficient to fully assess all the costs and benefits of PPA CMR. Improvements in risk profile will only translate in a reduction in cardiometabolic events in the longer term. Modeling is therefore necessary to extrapolate study findings to the longer term. The RIVM-CDM, developed at the National Institute for Public Health and the Environment, has been widely accepted for evaluation of cost-effectiveness, also in other prevention programs [34,36-38]. A disadvantage of modeling is the potentially large effect of small uncertainties of input data on the output of the model. For instance, if the effect of PPA CMR on patients’ risk profiles would decrease after one year, this could result in an overestimation of the long-term effects of the program. Probabilistic sensitivity analysis will be performed to assess the level of uncertainty of model outputs.

### Non-response analyses

The results of the non-response analyses of the INTEGRATE study will provide more information about the characteristics and motives of non-participants in PPA CMR. This knowledge is relevant and essential for the development and evaluation of participation enhancing strategies. The INTEGRATE study has a unique design where the results of the non-response analyses, performed at an early time point during the study, can be used as input for developing interventions to increase the participation rate later in the study. Effective participation enhancing strategies are useful when optimizing implementation of future prevention programs in primary care.

In comparable studies, including the pilot implementation of PC [14,15] the response rates were low, ranging from 3% to 75% [14,15,18]. Since this has been taken into account in the sample size calculation, sufficient power is expected even with low response rates. To enhance participation rates we plan to use several strategies, based on advise and results of previous studies [14] and on non-response analyses during the study. The accompanying information letter will emphasize safety in handling privacy sensitive data, especially digital data. Furthermore, the information letter will contain a short recap of the purpose of the letter and the advice to ask a family member for help with translation if considered necessary. The letter will present the recap in different languages. Reminder letters with a paper version of the risk estimation will be sent to all non-responders after two weeks. Furthermore, we evaluate a subgroup that is offered the possibility to bypass one of the GP consultations by ordering a toolbox. The toolbox is a tool that stimulates self-management; patients are able to take more responsibility for their own health. Furthermore, obtaining the additional measurements through a toolbox is easier to incorporate into ones busy life and this might enhance participation rates. A higher participation rate increases the cost-effectiveness of the entire program.

### Possible methodological threats

Several measures minimize possible bias in this study. To prevent selection bias, we aim at a representative sample for all GP practices in the Netherlands. Participating practices will be balanced in urban and rural locations and will have variable sizes, containing both solo and group practices. Selective participation can be an issue, since prevention programs sometimes tend to attract the patients referred to as the ‘worried-well’ [14,18,39]. However, the pilot implementation of the prevention program PC showed no presence of this effect [14,15]. The non-response analysis performed during study is sensitive to selection bias in case of low response rates and selective responders.

During this study participants are asked to report their own expenses and health care utilization, including consultations. Data collection by self-report can induce recall bias, but in combination with EMR data, we assume the outcome measures to be more reliable.

### Implementation challenges

Due to health care policy there is a possibility that changes in the health care environment will occur over time. For example, changes in established compensations for participation in prevention programs by health care insurers can influence the compliance and participation rates. However, these changes will occur in both the intervention groups and the waiting list control groups equally, so we expect this will not influence our study results.

### Conclusion

Prevention programs for CMD are an actual topic in health care. Under pressure of politics and society, implementation of these programs has already been initiated. Nevertheless, primary prevention of CMD by early risk factor modification has not yet been proven effective and cost-effective at population level. Before implementation on a large scale can be carried out, scientific support must be presented. If the INTEGRATE study shows PPA CMR to be effective and cost-effective, this will provide the evidence base that is needed for setting up prevention programs for CMD at national level. With determination of the profile of non-responders in prevention programs in primary care, the results of the INTEGRATE study will also assist in the development and implementation of similar prevention programs.

## Abbreviations

CMD: Cardiometabolic disease; EMR: Electronic medical record; GPs: General practitioners; HDL: High-density lipoprotein; ICER: Incremental cost-effectiveness ratio or incremental cost-utility ratio; ICPC: International classification of primary care; N: Number; PC: Preventive Consultation (Dutch: PreventieConsult CardioMetaboolRisico); PPA CMR: Personalized prevention approach for cardiometabolic risk; QALY: Quality-adjusted Life Years; RIVM-CDM: National Institute of Public Health and the Environment (RIVM) – Chronic Disease Model; SES: Socio-economic status.

## Competing interests

All authors declare that they have no competing interests.

## Authors’ contributions

MN, MH, FS and NW wrote the study proposal and obtained funding for the study. IB and DS wrote the final manuscript for the study protocol. IB, DS, MN and MH will conduct the study. FS, NW, RK, GW participate in the coordination and supervision of the study. The manuscript for the study protocol was discussed, edited and revised by all authors. All authors read and approved the final manuscript.

## Authors’ information

Ilse F Badenbroek and Daphne M Stol are co-first authors.

## Pre-publication history

The pre-publication history for this paper can be accessed here:

http://www.biomedcentral.com/1471-2296/15/90/prepub
