# Erratum to: Design of the INTEGRATE study: effectiveness and cost-effectiveness of a cardiometabolic risk assessment and treatment program integrated in primary care

**DOI:** 10.1186/s12875-016-0438-7

**Published:** 2016-04-06

**Authors:** Ilse F Badenbroek, Daphne M Stol, Marcus MJ Nielen, Monika Hollander, Roderik A Kraaijenhagen, G Ardine de Wit, François G Schellevis, Niek J de Wit

**Affiliations:** Netherlands Institute for Health Services Research (NIVEL), P.O. Box 1568, Utrecht, 3500 BN The Netherlands; Julius Center, University Medical Center Utrecht, Utrecht, The Netherlands; NDDO Institute for Prevention and Early Diagnostics (NIPED), Amsterdam, The Netherlands; Centre for Nutrition, Prevention and Health Care, National Institute of Public Health and the Environment (RIVM), Bilthoven, The Netherlands; Department of General Practice & Elderly Care Medicine/EMGO Institute for health and care research, VU University Medical Center, Amsterdam, The Netherlands

## Erratum

### Background

The INTEGRATE study investigates the effectiveness and cost-effectiveness of a “Personalized Prevention Approach for Cardiometabolic risk” (PPA CMR). This is a combination of an online risk estimation as used in the Dutch guideline ‘the Prevention consultation’ (Dutch PC-guideline) [1] and a tailored lifestyle intervention. The different steps of PPA CMR are described in our protocol [2].

### Interim analysis

#### First results INTEGRATE

The first interim analysis in October 2014 in 11 practices showed expected response rates of 40 % on the first step. However, the results of the online risk estimation (step 2) were different than expected. Only 27 % of the participants had a score above threshold and was eligible for the third step. This is far less than the 60 % that we had expected, based on results of the pilot study in 2009 [3]. As a consequence, only half of the expected participants proceeds to step 3 of the intervention (additional measurements).

#### Risk estimation

The explanation for the difference between the findings is a slight change in the algorithm of the risk score used for the 2011 Dutch PC-guideline as compared to the algorithm used in the 2009 pilot study. According to information provided by the guideline team of Dutch College of GPs, responsible for the guideline, the risk score calculation was reassessed before publication in the Dutch PC-guideline.

The assumptions made for the sample size calculation for the INTEGRATE study are based on the results of the risk score calculation in the pilot study.

The guideline authors and the INTEGRATE research team conclude that there is a chance that the risk score calculation as used in the INTEGRATE study could lead to a number of misclassified participants at moderate risk for cardiometabolic diseases (CMD) who score under the threshold. To study this, we have decided to adapt the study protocol.

### Amendment in protocol

In addition to our published protocol we will perform additional measurements in a selection of participants with scores below threshold in April and June 2016. We will invite this group for the same intervention as the participants with a score above threshold.

Criteria for inviting people for additional measurements will be participants with one of the following risk factors for CMD:

- a family history of cardiovascular disease
- BMI >27
- smokers aged 50 and older

The results will show the number of newly detected CMD and CMD risk factors in a subgroup of participants with scores below threshold. Sensitivity analyses will show in what range the risk estimation is most (cost-) effective. Based on these results we will be able to give advice whether to reassess the threshold of the risk score in the Dutch PC guideline.

#### Consequences

- The aim of the study remains unchanged: “*the effectiveness and cost-effectiveness of a cardiometabolic risk assessment and treatment program integrated in primary care*”.
- The sample size calculation is no longer applicable. The intervention group will be smaller than expected in the original protocol. This has consequences for the power of the study. The study might not have sufficient power to detect a difference in the number of smokers. However, the study will have sufficient power to detect differences in the other CMD risk factors such as BMI and blood pressure.
- The cost-effectiveness analysis will be performed according to plan
- Additional measurements will be performed in the last two groups of study participants in April and June 2016 (eligible participants n = 10.000) with risk scores below threshold and aforementioned risk factors for CMD.

### Ethics and funding bodies

The described amendment in our protocol was approved by the UMC Utrecht Institutional Review Board and exempted from full assessment under the Medical Research involving human subjects Act.

We have received additional funding by ZonMw (The Netherlands organization for Health Research and development), Lekker Lang Leven (a collaboration of the Dutch Diabetes Research Foundation, the Dutch Heart Foundation and the Dutch Kidney foundation) and Innovatiefonds Zorgverzekeraars (Healthcare Insurance Innovation Fund) to compensate for the 6 month delay and the costs for the additional measurements. The Dutch College of GPs who developed the Dutch PC-guideline fully supports the amendment made in our protocol.

## Conclusion

The amendment in the protocol is in our opinion the best solution to guarantee the validity of the INTEGRATE study. The aim of our study remains unchanged. However, the amendment will enable us to establish the optimal and most cost-effective threshold for the online risk estimation. Furthermore it gives us the opportunity to advice the Dutch College of GP’s how to improve the Dutch PC-guideline.
